# Transmembrane voltage potential of somatic cells controls oncogene-mediated tumorigenesis at long-range

**DOI:** 10.18632/oncotarget.1935

**Published:** 2014-05-01

**Authors:** Brook T. Chernet, Michael Levin

**Affiliations:** ^1^ Center for Regenerative and Developmental Biology and Department of Biology Tufts University 200 Boston Avenue,Suite 4600 Medford, MA 02155 U.S.A.

**Keywords:** cancer, tumors, ion channels, bioelectricity, resting potential, V_mem_, transmembrane potential, HDAC, butyrate, microenvironment, bacteria

## Abstract

The microenvironment is increasingly recognized as a crucial aspect of cancer. In contrast and complement to the field's focus on biochemical factors and extracellular matrix, we characterize a novel aspect of host:tumor interaction – endogenous bioelectric signals among non-excitable somatic cells. Extending prior work focused on the bioelectric state of cancer cells themselves, we show for the first time that the resting potentials of distant cells are critical for oncogene-dependent tumorigenesis. In the *Xenopus laevis* tadpole model, we used human oncogenes such as mutant KRAS to drive formation of tumor-like structures that exhibited overproliferation, increased nuclear size, hypoxia, acidity, and leukocyte attraction. Remarkably, misexpression of hyperpolarizing ion channels at distant sites within the tadpole significantly reduced the incidence of these tumors. The suppression of tumorigenesis could also be achieved by hyperpolarization using native CLIC1 chloride channels, suggesting a treatment modality not requiring gene therapy. Using a dominant negative approach, we implicate HDAC1 as the mechanism by which resting potential changes affect downstream cell behaviors. Based on published data on the voltage-mediated changes of butyrate flux through the SLC5A8 transporter, we present a model linking resting potentials of host cells to the ability of oncogenes to initiate tumorigenesis. Antibiotic data suggest that the relevant butyrate is generated by a native bacterial species, identifying a novel link between the microbiome and cancer that is mediated by alterations in bioelectric signaling.

## INTRODUCTION

Normal embryonic development, as well as repair and dynamic maintenance of complex structures throughout the lifespan, both depends upon a set of signals that keeps individual cell activities orchestrated toward the large-scale anatomical goals of the host. Morphogenesis and remodeling can be challenged by cancer, which can be viewed as a process in which cells escape or become isolated from the normally tight morphogenetic control of the organism [1-6]. To achieve robust development, signaling pathways need to affect their target cells with sufficient spatiotemporal resolution to integrate organ sculpting, anatomical polarity, tissue identity, and growth rates appropriate to the large-scale order maintenance within the body. In the context of cancer, it has been well documented that healthy neighboring cells help to stabilize aberrant cell behavior [7-9] to control tumorigenesis by tissue-level organization that adheres to the proper patterning needs of the host [10-14]. Indeed, fascinating classical and recent data show that actively patterning environments, such as embryos and regenerating amphibian limbs, can normalize and reprogram tumors [15-31]. Thus, in addition to any cell-autonomous properties that may have gone awry in cancer cells, it is crucial to understand the non-cell-autonomous patterning signals that may be exploited to prevent and treat cancer.

Endogenous bioelectric signaling among all (not just excitable) cell types is one component of the microenvironment, and is now known to mediate instructive information for large-scale pattern formation [32, 33]. Bioelectrical processes, such as extracellular electric fields and transmembrane resting potentials produced by ion channel and pump proteins, regulate cell activity [34-36]. In particular, proliferation, differentiation, migration, and apoptosis are all important cell behaviors relevant to cancer that are guided partly by bioelectric signals, such as resting voltage potential (V_mem_) in non-excitable cells [37-39]. It was recognized long ago that the electrical properties of mature tumors differ from that of healthy tissue [40-42], and we recently showed that monitoring of a distinctly depolarized resting potential allows early detection of tumors *in vivo*. Moreover, forced hyperpolarization via a number of different channels prevents those cells from forming tumors [43], revealing resting potential as an instructive cue and not merely a marker [39]. Indeed, a number of ion channels are now known to be *bona fide* oncogenes [44-54], and ion channel blockers are an important area for cancer drug development [55-58].

However, it is important to note that ion channels are not simply molecular markers or targets within the cancer cells themselves. Gradients of resting potential are a long-range, global system for exerting patterning control, and thus a tractable candidate for manipulation of the crosstalk by which microenvironment suppresses aberrant cell behavior [59]. For example, selective depolarization of glycine-gated chloride channel-expressing cells in the *Xenopus* model results in a metastatic conversion of melanocytes, which over-proliferate, acquire an arborized shape, and invade blood vessels and soft tissues in an MMP-dependent manner [46, 60, 61]. This effect occurs at considerable distance, via a serotonergic pathway, and demonstrates how disruption of long-range endogenous physiological signaling can activate a cancer phenotype in the absence of carcinogen exposure or DNA damage. The data suggest that bioelectric gradients are a fascinating new aspect of cancer:host interaction, and that the notion of microenvironment may need to be expanded to account for long-range interactions mediated by V_mem_ changes throughout tissues.

The existing literature on roles of ionic signaling in cancer leaves open a number of fundamental questions. What role might remote V_mem_ modulation play in oncogene-induced carcinogenesis? While distant changes of resting potential can trigger a metastatic conversion [60, 61], it is not known whether or how such signaling plays a role when canonical oncogenes initiate tumorigenesis. A better understanding of the microenvironment and long-range aspects of aberrant growth control by endogenous developmental patterning mechanisms would have significant implications for design of novel therapeutic approaches to prevent and reprogram cancer.

Here, we use mRNA encoding human tumor inducers (*Gli1, XRel3, KRAS*) in *Xenopus laevis* embryos to initiate growth of tumor like structures (ITLS) that highly resemble classic tumors. Remarkably, forced hyperpolarization (by misexpression of specific ion channels) is able to suppress ITLS formation, despite high levels of oncogene protein, even when the hyperpolarized cells are at a considerable distance from the oncogene-expressing tumor site. The suppression effect can also be exerted by native chloride intracellular channel 1 (CLIC1)-mediated hyperpolarization, revealing an endogenous target for bioelectric control of abnormal growth that does not require transgene expression. We show that the suppression effects of distant hyperpolarization are mediated by voltage control of a butyrate and histone deacetylase mechanism. Together, these data reveal the first mechanistic details of V_mem_ as a powerful, tractable regulator of long-range signaling between cancer cells and the host and suggest several new entry points for biomedical strategies.

## RESULTS

### Induced tumor-like structures (ITLS) exhibit striking key similarities to human tumors

To study the role of bioelectric events to oncogene-mediated tumorigenesis, we took advantage of *Xenopus* laevis embryos – a model system that is ideal for molecular biophysics approaches and also has been increasingly used for cancer research[43, 61-66]. We injected mRNAs encoding Xrel3 and human *KRAS*^G12D^ into *Xenopus* embryos; these oncogenes are known to cause morphologically apparent ITLS in up to 50% of injected embryos without any other developmental defects. ITLS have previously been characterized as having some of the hallmarks of human tumors – increased proliferation, vasculature attraction, lack of differentiation, invasiveness and transplantability to healthy recipients [43, 67-69]. We began by further investigating the pathology of the affected tissue to confirm and expand the relevance of *Xenopus* as a medically-relevant cancer model.

**Figure 1:**
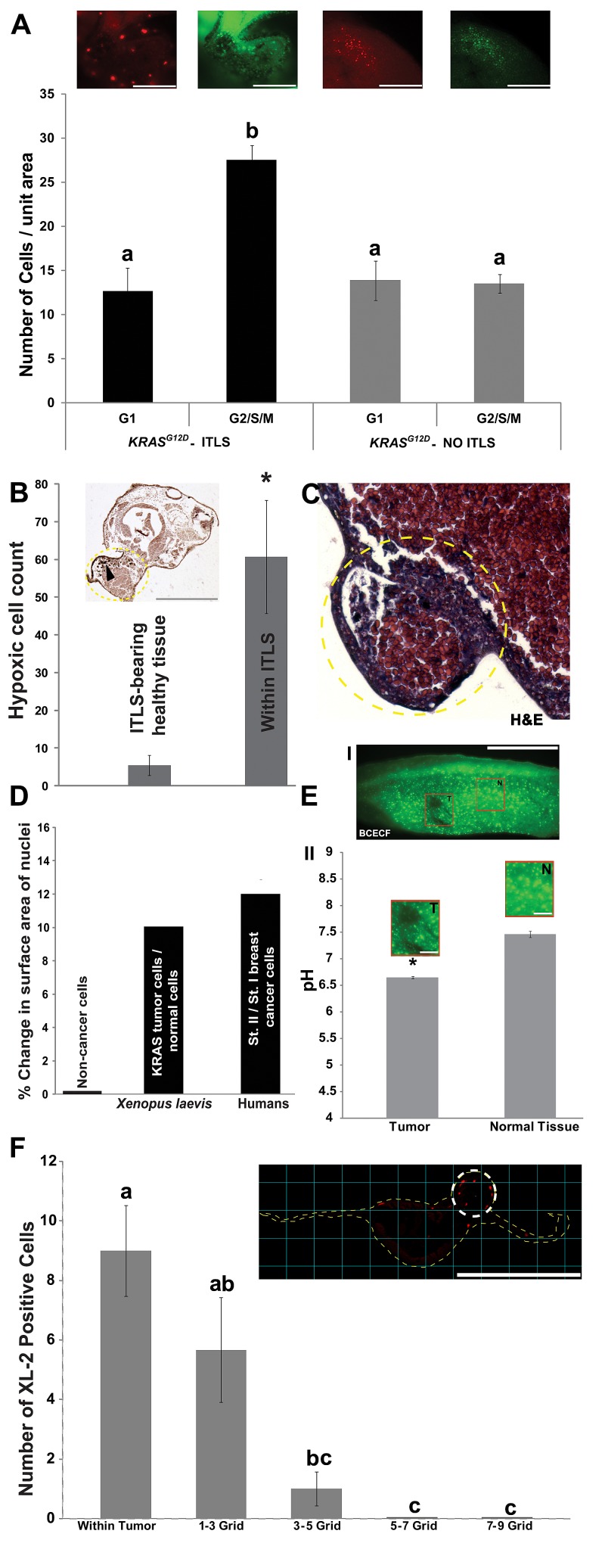
Induced tumor-like structures (ITLS) exhibit characteristics reminiscent of human tumors (A) Rates of proliferation were analyzed *in vivo* in fluorescent cell cycle indicators (FUCCI pair: mKO2-zCdt1 and mAG-zGeminin) injected, ITLS-bearing embryos at stage 34. ITLS regions (black bars) have >65% more cells that are in G2/S/M phase (green insert, mAG-zGeminin) than there are in G1 phase (red insert, mKO2-zCdt1). Unperturbed regions (grey bars) showed no difference between the number of cells in G1 (red insert) and G2/S/M (green insert) phases. *N*=8 for all four categories. *P*<0.001, one-way ANOVA, tukey's post hoc analysis; different letters indicate statistically significant difference; scale bar = 250 µm.(B) Immunoperoxidase analysis of hypoxia using detection of pimonidazole protein adducts (black arrowhead) in cells reveals a 12 fold increase for immunoperoxidase staining for hypoxia per unit area in ITLS (yellow traces) than in surrounding healthy tissue. *N*=7; *P<0.0001 Student's *t*-test; scale bar = 500 µm.(C) H&E staining of *KRAS*^G12D^ ITLS sections (yellow circular trace, 40X) for evidence of features of neoplasia reveals disorganized growth patterns, including misplaced mesodermal and endodermal cells, and mesodermal cells that are larger than those present in unperturbed regions. (D) Automated analysis of the size of Hoechst Blue stained nuclei reveals a 10% increase in the size of tumor nuclei compared to nuclei from unaffected cells. A change in morpholology of the nuclei that includes a progressive increase in nucleus size has been documented in human breast cancer cells [82]. *N*=16 (2371 nuclei) for both ITLS and unaffected regions.(E) Intracellular pH measurements in ITLS and Control regions were made using the fluorescent pH reporter dye BCECF. (I) A decreased fluorescence is observed in ITLS cells (T) compared to a normal region (N). (II) Upon quantification, levels of fluorescence correspond to pH values of 6.6 in ITLS and 7.41 in control regions. *N*=8 for both treatments; *P<0.05 Student's *t*-test; scale bar = 1mm in tail fragment; 150 µm in magnified inserts.(F) The response of innate immunity to ITLS formation was investigated using anti-XL2 immunohistochemistry to mark the presence of leukocytes: leukocytes are primarily present around ITLS (white circular trace) and 1-3 grid (300 µM) away from ITLSs. *N*=12; *P*<0.01, one-way ANOVA, Tukey's post hoc analysis; different letters indicate statistically significant difference; scale bar = 500 µm. Error bars indicate ± 1 s.e.m in A, B, E, F.

Clinically-relevant tumors exhibit a surplus of proliferative capacity [70]. To characterize the cell proliferation dynamics in ITLS loci, we monitored dynamic patterns of cell cycle progression in ITLS and unaffected regions using FUCCI (*f*luorescent *u*biquitination-based *c*ell *c*ycle *i*ndicator) pair: mKO2-Cdt1 and mAG-Geminin [71, 72]. The accumulation of these two cell cycle regulators – in nuclei of transfected cells – in a mutually exclusive manner between G1(Cdt1) and G2/S/M (Geminin) phases, allowed us to monitor spatial dynamics of cell cycle progression: while the number of ITLS cells in G1 and control cells in G1 and G2/S/M phases are statistically the same, twice as many cells in G2/S/M phases were present in ITLS by comparison (*N*=8 per category, ANOVA, *P<0.001*) (Fig. 1A).

Next, we examined the extent of hypoxia within ITLS loci, since hypoxia is known to be present in tumors [73-75]. Treatment of ITLS bearing embryos (*N*=7) with the hypoxia marker pimonidazole revealed a 12-fold increase (*t*-test; p<0.0001) for immunoperoxidase staining for hypoxia (Fig. 1B). This is consistent with data obtained from pimonidazole-based qualitative and quantitative assessment of tumor hypoxia, where an increase in the frequency of detecting pimonidazole adducts is reported [76-78].

We next examined histological features of the ITLS using hematoxylin and eosin (H&E) [79]. Cells within the ITLS exhibited disorganized growth patterns, including misplaced mesodermal and endodermal cells, and mesodermal cells that were larger than those present in unperturbed regions, revealing features consistent with neoplasia (Fig. 1C).

We next examined a change in nuclear size, which is another well-known aspect of tumor tissue [80, 81]. Computer-aided image analysis of the area of 2371 nuclei each from 16 ITLS and 16 control regions of oncogene-injected tadpoles showed on average a 10% increase in nuclear size of ITLS, mirroring similar phenomenon observed between different stages of human breast cancer cells [82] (Fig. 1D). Another marker of tumorigenesis is the abnormal acidity of tumor tissue [83, 84]. Using BCECF, a dual excitation pH indicator, fluorescence intensity ratios at two different wavelengths of ITLS and control regions revealed that ITLS have a more acidic (pH 6.6) intracellular environment compared to that of control regions, which have a pH average of 7.41 (*N*=8 for both treatments, *t*-test, *P<0.05*) (Fig. 1E).

Finally, to investigate host immune response to tumor formation, immunohistochemistry with anti-XL2 (anti-*Xenopus* leukocytes) was performed on cross sections of tissue bearing ITLS. We observed recruitment of leukocytes to the site of transformed cells (Fig 1F), consistent with the known role of leukocyte migration as an anti-tumor immune response [85].

Taken together, these characteristics of ITLS are consistent with previous findings of increased proliferation within ITLS, ability to form in internal tissues (not just the epidermis), and propensity to attract vasculature[43], and demonstrate the relevance of human oncogene-induced tumors in *Xenopus* for understanding the basic mechanisms of carcinogenesis *in vivo*.

### Long-range hyperpolarization suppresses the formation of ITLS

**Figure 2:**
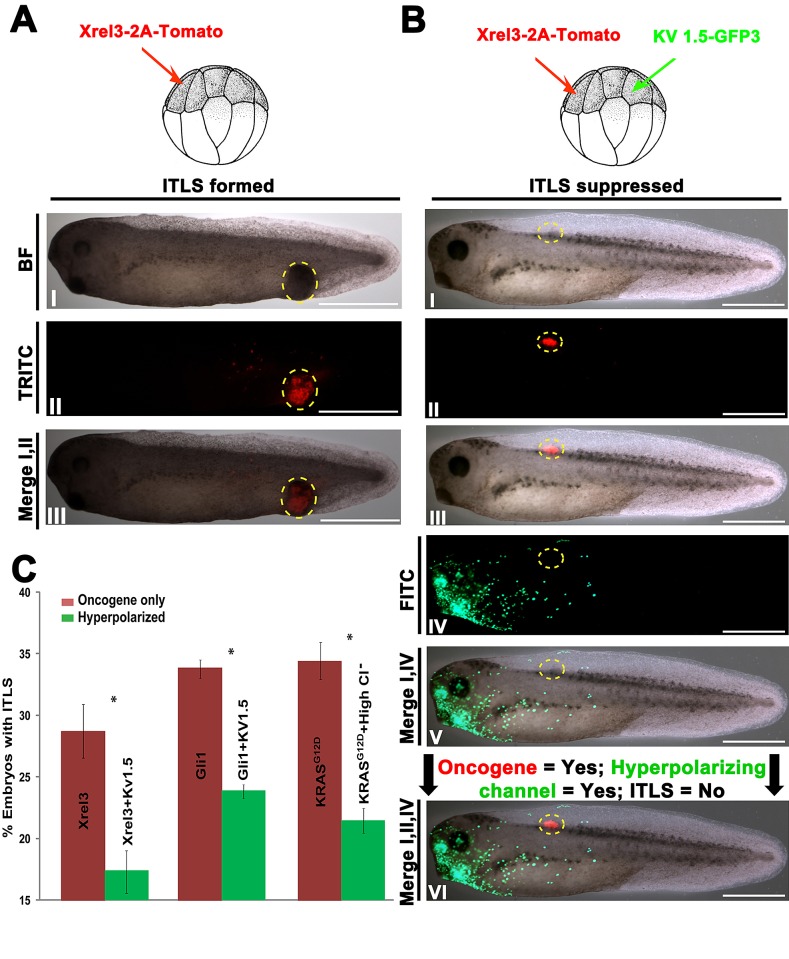
Long-range hyperpolarization suppresses the formation ITLSs (A) To visualize and track ITLSs, mRNA encoding a fusion construct of *Xrel3* and tdTomato was injected into a single blastomere of 16-cell stage embryos. (I, II) ITLSs resulting from *Xrel3*-2A-tdTomato injections were highly fluorescent when visualized under a TRITC filter set. (III) Overlay of bright field (BF) and TRITC images shows the co-localization of ITLS and red fluorescent signal, confirming that foci of oncogene expression are the cells that make up ITLS. Scale bar = 1 mm.(B) To test whether long-range hyperpolarization suppresses ITLS formation, a potassium based hyperpolarizing channel (Kv1.5-GFP3) and *Xrel3*-2A-tdTomato were injected in distantly separated blastomeres of a 16-cell stage embryo. (I) While morphologically-apparent ITLSs were often missing from these embryos, the oncogenic protein was present as evidenced by the tdTomato fluorescent signal (II, II). Robust expression of the hyperpolarizing channel protein (Kv1.5-GFP3) was observed in the head and gut regions (IV, V), and away from Xrel3-2A-tdTomato expressing cells (VI). Scale bar = 1 mm.(C) Fold change in ITLS formation for oncogene injected embryos with hyperpolarized treatments compared to oncogene-only injected embryos. Oncogene-only injected embryos with ITLS have a ratio of 1, and ratio values below and above one represent fewer and more embryos with ITLS, respectively. To show the effect of ITLS suppression due to change in Vmem, three different oncogenes and hyperpolarizing reagents (based on Cl^−^ and K^+^) were used. When Kv1.5 (K^+^ hyperpolarizing channel) was introduced non-locally in *Xrel3* and *Gli1* injected embryos, 39.4% and 29.4% decreases, respectively, in ITLS formation were observed. A 37.5% decrease in ITLS formation was also achieved using *KRAS*^G12D^ as an oncogene and 70mM Cl^−^ as a hyperpolarizing reagent. *N*=225-349 embryos for each treatment; *P<0.01 Student's t-test. Error bars indicate ± 1 s.e.m.

To test the hypothesis that hyperpolarization affects oncogenic transformation of cells located at a distance, we performed functional experiments using sets of oncogenes and hyperpolarizing reagents. The use of multiple oncogenes and hyperpolarizing agents allowed us to confirm that there is a generalized suppression effect due to changes in V_mem_, one that is not tied to a particular channel protein or ion. We used *Xrel3* tagged with tdTomato to track the presence of oncogenic protein (Fig. 2A, I-III). To modulate V_mem_, we used Kv1.5 (Potassium voltage-gated channel, shaker-related subfamily, member 5) [86], a well-characterized hyperpolarizing channel, whose overexpression in our model system has been used to alter developmental patterning [87].

To probe the effects of long-range hyperpolarization on tumor formation, we injected mRNA's encoding Xrel3-tdTomato and Kv1.5-GFP3 in two separate blastomeres of 16-cell stage embryos, and scored for the presence of ITLS and hyperpolarizing channel/oncogene protein by stage 34. Compared to embryos receiving only oncogene injections, embryos with a remote hyperpolarized region (Fig. 2B, IV&V) often displayed lack of morphologically apparent ITLS (Fig. 2B, I) despite strong presence Xrel3-2A-tdTomato protein (Fig. 2B, II&III). Kv1.5-induced long-distance hyperpolarization was also able to significantly suppress *Xrel3* and *Gli1* ITLS formation by 34.9% (*N*=225; *t*-test, *P*<0.05) and 29.4% (*N*=292; *t*-test, *P*<0.05), respectively (Fig. 2C), suggesting the effect is not specific for just one type of oncogene. Treatment with high chloride (whose uptake by native Cl^−^ channels hyperpolarizes cells) resulted in a 37.5% decrease (*N*=349; *t*-test *P<0.05*) in *KRAS*^G12D^ ITLS, demonstrating that the suppressive effect is not specific for Kv1.5 nor for potassium, as mediators of the crucial V_mem_ change (Fig. 2C).

Collectively, these data reveal a long-range signal that is initiated by hyperpolarization and has influence over tumorigenesis at remote sites in the tadpole. Moreover, these results demonstrate that ITLS suppression through V_mem_ modulation is possible to achieve by taking advantage of endogenous channels that are not necessarily located within oncogene expressing cells.

### Chloride-dependent hyperpolarization through endogenous chloride intracellular channel 1 (CLIC1)

**Figure 3:**
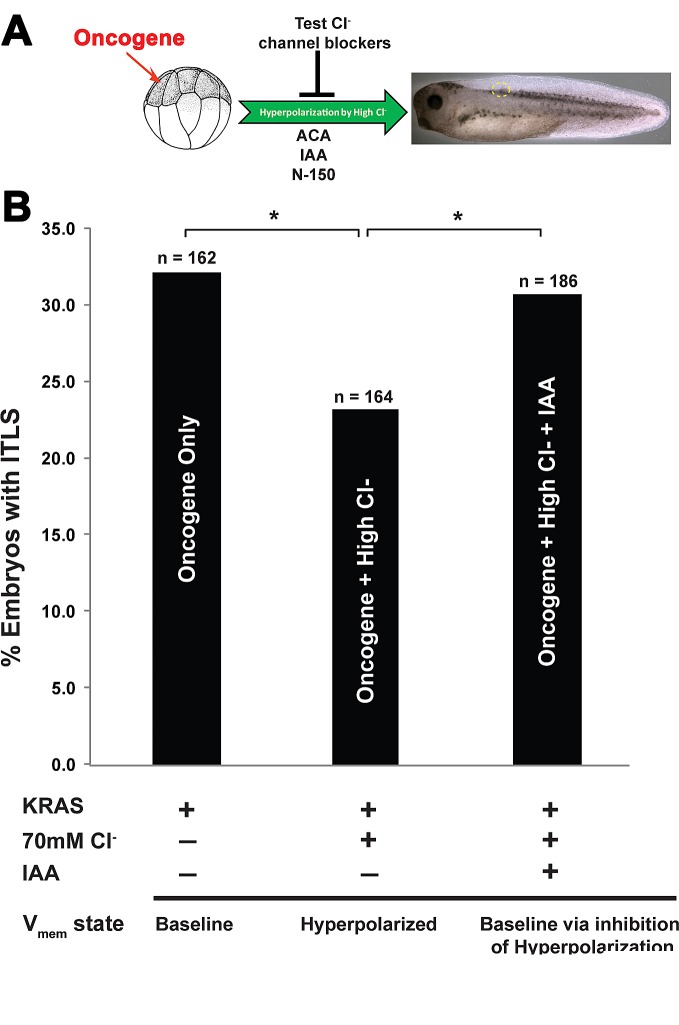
A suppression screen for chloride channels is employed to identify a channel responsible for long-range ITLS suppression (A) Oncogene injected embryos were placed in a high chloride concentration (70mM) solution, followed immediately by separate treatments with very well-known chloride transport blockers: Anthracene-9-Carboxylic Acid (ACA), indanyloxyacetic acid (IAA) and 5-Nitro-2-(3-phenylpropylamino) benzoic acid (NPPB). (B) High chloride treatment of oncogene-injected embryos results in ~31%% decrease in *KRAS*^G12D^ ITLS incidence. While blocking chloride channels using ACA and NPPB did not affect the rate of this suppression, IAA treatments in the presence of high Cl^−^ restored ITLS incidence back to oncogene-only levels. *N*=162, 164, 186 for oncogene only, oncogene + high Cl^−^, and oncogene + high Cl^−^ + IAA, respectively; *P<0.05, χ^2^ compared to tumor incidence in *KRAS*^G12D^ only injected embryos.

Having shown a functional role of long-range hyperpolarization in the context of tumorigenesis, we proceeded to characterize the native channel responsible for chloride-based inhibition of ITLS formation. We performed an inverse drug screen [88] with chloride channel blockers to see which ones would abrogate the chloride-dependent ITLS suppression: Anthracene-9-Carboxylic Acid (ACA), inadyloxyacetic acid 94 (IAA), and 5-Nitro-2-(3-phenylpropylamino) benzoic acid (NPPB) [89-92] (Fig. 3A). After the injection of *KRAS*^G12D^ and treatment with high chloride (*N*=164), ITLS incidence dropped significantly (χ^2^, *P*<0.05) compared to their counterparts in a low chloride concentration (*N*=162). This anti-tumor effect of high chloride was completely abolished with IAA treatment (N=186; χ^2^, *P*<0.05) (Fig 3B), while the other two inhibitors did not significantly affect the rate of ITLS incidence. We then proceeded to validate this result using molecular reagents targeting IAA-sensitive channels.

**Figure 4:**
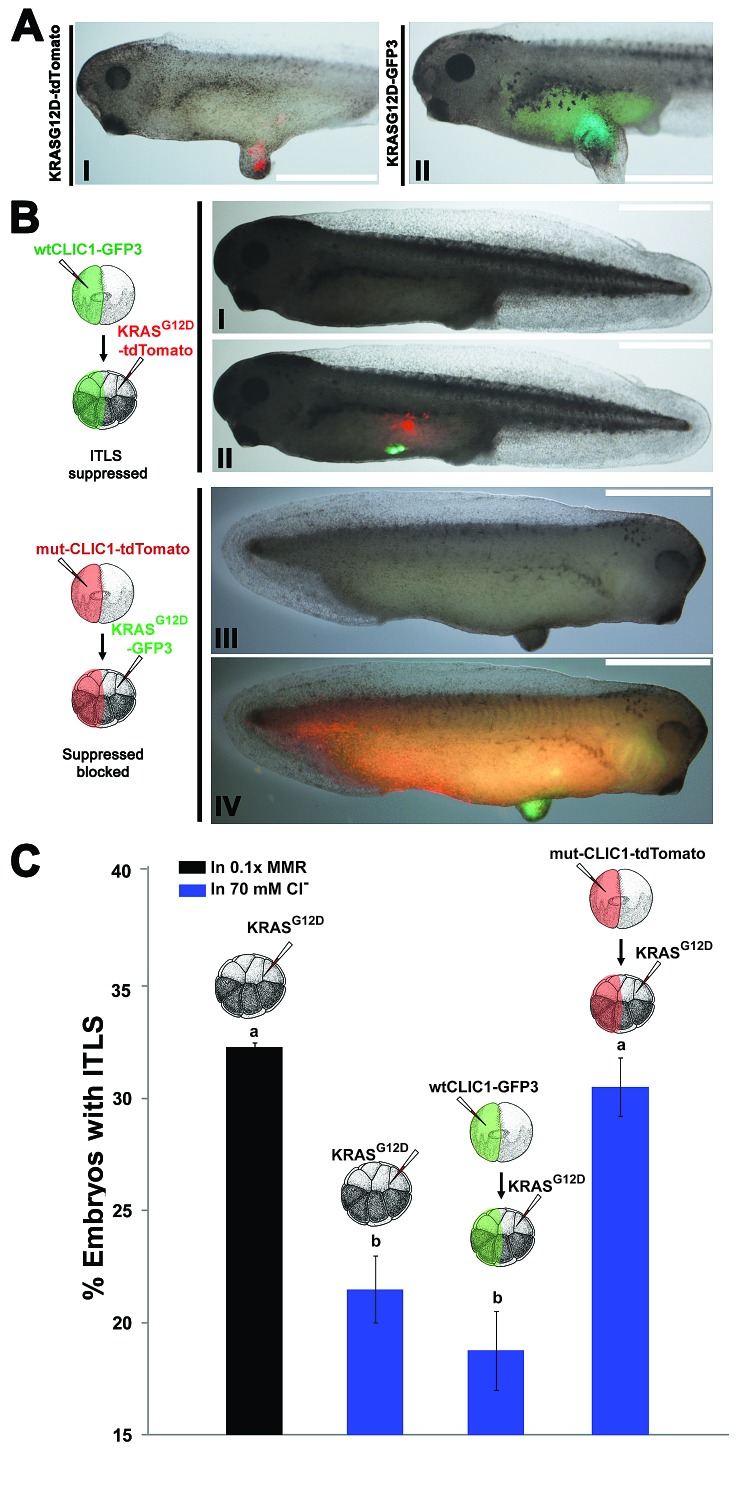
Hyperpolarization by the influx of Cl- through CLIC1 mediates long-range ITLS suppression (A) To visualize and track ITLSs, *KRAS*^G12D^ mRNA was co-injected with GFP3 (I) and tdTomato (II) into a single blastomere of 16-cell stage embryos. In both cases, ITLS co-localize with the respective fluorescent signals, confirming that oncogene-expressing cells make up ITLS. Scale bar = 1 mm.(B) When mRNA encoding *Xenopus* wildtype CLIC1-GFP3 was injected at a distance from *KRAS*^G12D^ -tdTomato, morphologically-apparent ITLS formation was often suppressed despite the presence of oncogenic protein (III). Whereas overexpression of a CLIC1 dominant negative (CLIC1C24S-tdTomato) in non-oncogene expressing cells prevents ITLS suppression despite the presence of high Cl^−^ in the media (IV). Scale bar = 1 mm. (C) Changes in ITLS formation incidence for oncogene and CLIC1 (mutant or wildtype) injected embryos in 70mM Cl^−^ media. ITLS incidence from oncogene-only injection was normalized to 1 so that it can be used to measure against the effects of other treatments. Embryos injected with *KRAS*^G12D^ followed by a high Cl^−^ treatment showed a 31.5% decrease in ITLS incidence. Overexpression of the wildtype CLIC1 and high Cl^−^ bath also lowered the number of embryos with ITLS by 44.3% while resulting in smaller ITLS in escapees. Overexpression of the mutant CLIC1, which encodes for defective multimer channels, appeared to block ITLS suppression by a high Cl- treatment, implicating native CLIC1 channels in Vmem mediated, long-range suppression of ITLS. N= 336-364 embryos for each treatment; P=0.01, one-way ANOVA, Tukey's post hoc analysis; different letters indicate statistically significant difference. Error bars indicate ± 1 s.e.m.

A known target of IAA is the intracellular chloride channel 1 (CLIC1) [93]. Given its pharmacological profile and ubiquitous presence during every stage of the *Xenopus laevis* development [94], we focused on CLIC1 in this study. To test the hypothesis that CLIC1-mediated hyperpolarization affects oncogenic transformation of cells, we performed functional experiments involving CLIC1 (wildtype and mutant) and *KRAS*^G12D^ injections. Wildtype CLIC1 was tagged with GFP, while a dominant negative form of CLIC1 was generated by mutating cysteine 24 to serine, in order to form an inactive multimer channel [95], and tagged it with tdTomato to track localization of the protein product. mRNA's encoding *CLIC1-2A-GFP3/KRAS*^G12D^-*tdTomato* (Fig. 4B, I&II) and *CLIC1C24S-2A-tdTomato/KRAS*^G12D^-*GFP3* (Fig. 4B, III&IV) were injected in two different blastomeres of 16-cell stage embryos, treated with high Cl^−^, and scored for the presence of ITLS and CLIC1 channel/oncogene proteins. As a control to which CLIC injected embryos can be compared, *KRAS*^G12D^-only injected embryos were also treated with high Cl^−^ (*N*=372), resulting in 31.5% fold decrease in ITLS incidence when compared to *KRAS*^G12D^-only injected embryos (*N*=341). The rate of ITLS suppression was further amplified (44.3% suppression) when the wildtype CLIC1 was overexpressed non-locally in oncogene-injected embryos that were then raised in 70mM Cl^−^ (*N*=364). In contrast, overexpression of the mutant CLIC1 (*N*=336) effectively blocked ITLS suppression despite the presence of high Cl^−^ (Fig 4C). Together, these data suggest a role for native CLIC1 in mediating hyperpolarization-induced long-range ITLS suppression.

### Changes in V_mem_ are transduced via HDAC1-dependent mechanisms

**Figure 5:**
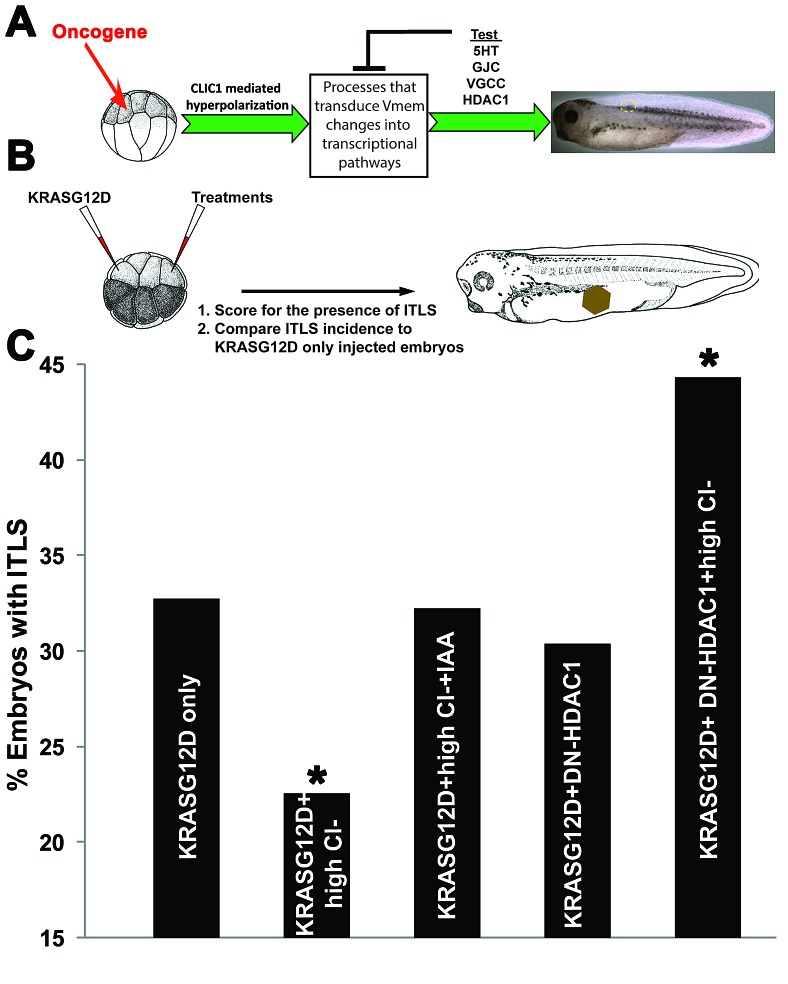
Long-range suppression of ITLS by hyperpolarization is HDAC1-dependent (A)Hyperpolarization can be transduced into transcriptional pathways by processes that include Vmem-dependent transport of signaling molecules (e.g. serotonin transport through SERT), electrophoresis of morphogenes through gap junctions (GJC), calcium signaling via voltage-gated Ca^++^ channels (VGCC), and voltage dependent butyrate transport that results in HDAC inhibition. To identify the responsible transduction mechanism, each process was independently blocked: 10 µM fluoxetine shuts down SERT; H7, a dominant connexin disrupts the transport function of endogenous gap junctions; 0.1 mM cadmium chloride effectively blocks VGCCs; and a dominant negative 0.5 ng HDAC1 injection inactivates endogenous HDAC1. (B) In each case, a treatment used to probe a given transduction mechanism was targeted non-local to *KRAS*^G12D^ injected cells. *KRAS*^G12D^ injections and treatments were administered at the 16-cell stage, and embryos were then raised to stage 34 before scoring the presence of ITLS.(C) When analyzing ITLS incidences compared to oncogene only injected embryos – DN-HDAC1blocks the effects of CLIC1 mediated hyperpolarization, thus brining ITLS formation to the level of oncogene-only injection. Introducing DN-HDAC1 at a distance from KRASG12D without hyperpolarization increases ITLS incidence by 35.6%, implicating a role for HDAC1 in long-range ITLS suppression. N=164-251 embryos; *P<0.05, χ2 compared to tumor incidence in KrasG12D only injected embryos.

Bioelectric signals are transduced into transcriptional and epigenetics responses via a number of second-messenger pathways. In order to determine the transduction mechanism by which hyperpolarization inhibits tumorigenesis, we conducted a pharmacological suppression screen [63] and molecular-genetic loss-of-function of several well-characterized candidate pathways that have been shown to mediate the actions of V_mem_ change in other patterning contexts [96]. Our strategy was to block, one at a time, the candidate transduction mechanisms and see which ones abrogated the ability of hyperpolarization to reduce tumor incidence. Targeted pathways include: movement of serotonin through the V_mem_-dependent serotonin transporter SERT [97] (inhibited via 10 µM fluoxetine); V_mem_-guided transport of signaling molecules through gap junctions [98, 99] (disrupted by 0.5 ng injection of a dominant negative connexin H7); voltage gated calcium channels [100, 101] (blocked via 0.1 mM cadmium chloride), and V_mem_-modulated transport of small molecule inhibitors of histone deacetylase 1 [102-104] (targeted by 0.5ng injection of mRNA encoding a dominant negative HDAC1) (Fig 5A). HDAC1 is an especially attractive target because it is known to control levels of histone acetylation, which determines cell cycle progression, rates of proliferation, apoptosis and differentiation of cancer cells [105-107]. Administering oncogene, hyperpolarizing agent, and V_mem_-sensing pathway disruptors (Fig 5B) showed that blocking HDAC1 activity had the most significant effect (χ^2^, *P*<0.05) on CLIC1-mediated suppression of tumors: the rate of ITLS formation in DN-HDAC1 injected embryos in high Cl^−^ was similar to that of oncogene-only injected embryos - introducing DN-HDAC1 non-locally to the site of *KRAS*^G12D^ increased ITLS formation incidence by 35.6% (χ^2^, *P*<0.05) (Fig. 5C), suggesting a role for histone deacetylase 1 activity controlling cell behavior at a distance.

**Figure 6:**
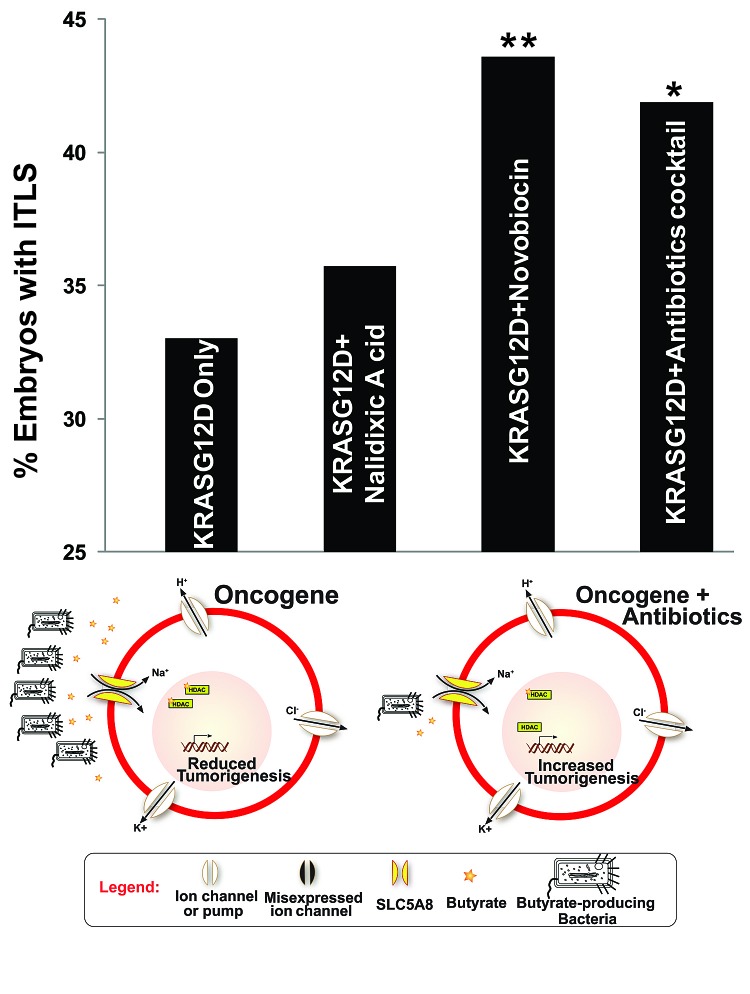
Targeted killing of butyrate producing Bacteria increases ITLS incidence To determine the source of butyrate, which is responsible for ITLS suppression through HDAC inhibition, variety of antibiotics were used to target butyrate-producing Bacteria Compared to KRASG12D-only injected embryos, novobiocin and antibiotic cocktail (gentamicin, metrodinazole, vancomycin, and clindamycin) treatments significantly increased ITLS formation by 32.1% and 26.8%, respectively. Adding nalidixic acid, which works by inhibiting DNA gyrase as novobiocin does, did not significantly alter ITLS formation, showing only an 8.2% increase in ITLS incidence. N=537, 318, 431, 117 for KRASG12D, KRASG12D + Nalidiixic Acid, KRASG12D + Novobiocin, KRASG12D + antibiotics cocktail; *P<0.05, **P<0.01, χ2 compared to ITLS incidence in KRASG12D-only injected embryos.

Having previously identified butyrate as an endogenous HDAC inhibitor involved in local ITLS suppression [43], we sought to identify the source of butyrate in this system. Reasoning that it would be produced by native bacteria, we conducted a screen for antibiotics that target butyrate producing bacteria. We first tested novobiocin, which has been shown to be effective against gram-positive bacteria [108]. At 78 μM, novobiocin sodium salt treatment significantly increased ITLS incidence by 32.1% (χ^2^, *P*<0.01) (Fig. 6). It has been determined in other studies that the mechanism of action for novobiocin involves the inhibition of the bacterial DNA gyrase [109, 110]. Given also nalidixic acid's well-known inhibition of DNA gyrase [111], we tested its effect (at 43 μM) on ITLS incidence. While an 8.2% increase in ITLS was observed, it was not statistically significantly different from ITLS incidence in oncogene-only injected embryos (Fig. 6). Finally, a cocktail of antibiotics (gentamicin, metrodinazole, vancomycin hydrochloride, and clindamycin hydrochloride, all at 20 μM concentration) targeting gram-positive bacteria resulted in a significant increase in ITLS incidence by 26.2% (χ^2^, *P*<0.05) (Fig. 6). Bulk analysis of embryos exposed to this cocktail of antibiotics does indeed show a significant drop in total butyrate concentration (data not shown).

Together, these data suggest that the control of tumorigenesis by V_mem_ is mediated by HDAC1, via butyrate derived from gram-positive bacteria.

## DISCUSSION

It has been well-documented that bioelectric properties control individual cell behaviors that are highly relevant to the processes of cancer [35, 36, 96, 112, 113]. For example, electric fields generated by voltage-gated sodium channels (VGSC) provide motility cues to highly metastatic rat prostate cells [114], and many cancer cells are galvanotactic [115-119]. Distinct from extracellular electric fields, resting membrane potentials established by ion channels and pumps control cell proliferation, transformation, and growth in number of tumor forming cells [120-125]. Moreover, the actions of these translocators have allowed for the development of predictive physiological markers [43, 61, 125] and control of proliferative and metastatic behaviors [39, 46, 60, 126]. Here, we show for the first time that resting potentials in remote cells have a profound influence on whether or not a cell expressing human oncogenes will go on to form a tumor *in vivo*.

The induced tumor like structures (ITLS) were shown to exhibit striking similarities with classic tumors, including increased proliferation as monitored by visualizing the cell cycle dynamics, disorganization of the normal developmental architecture revealed by H&E stains, increased hypoxia, increased nuclear size, acidic intracellular microenvironment, and ability to illicit innate immune response (Fig. 1A-F). Given the increased recognition of bioelectric signals as endogenous patterning cues that keep cellular activities orchestrated towards anatomical goals [32, 33, 127], our data confirm *Xenopus* embryos as a tractable model system in which to investigate the role of bioelectric signals in host:cancer interaction, and in which to ask fundamental questions about cancer as a disorder of developmental cues [5, 6, 10, 12, 128, 129].

### Long-range control of tumorigenic process by V_mem_

The spatial distance over which carcinogenesis can be predicted and controlled have been addressed in a few studies. One example is illustrated by the ability to assess cancer risk of readily-inaccessible organs using accessible surrogate sites – functionally or anatomically similar, but not necessarily contiguous with the target organ – that make up the cancer field [130-132]. Another is the activation of melanoma-like transformation in the melanocyte population [60] by V_mem_ modulation of even a few instructor cells at considerable distance from the melanocytes themselves [61]. However, to our knowledge this is the first time that modulation of distant cells' resting potentials has been shown to impact the formation of discrete tumor foci. It is tempting to speculate that the long-range connections are bi-directional: not only does remote electrical state of tissue matter for tumor growth, but perhaps tumors also emit bioelectrical information that could be detectable at a distance. This is compatible with classical data [131, 133-135] and will be tested using molecular reagents in the future.

In our previous work [43], we showed that hyperpolarized cells were themselves resistant to transformation [136-138]. To analyze long-range effects of hyperpolarization, we established an assay in which *Xenopus* 16-cell embryos were injected with oncogene in one blastomere and subjected to a hyperpolarizing treatment, non-locally, via ectopic-expression of hyperpolarization channel or manipulation of ionic contents of the media. Remarkably, the data show that hyperpolarization significantly suppresses the formation of ITLS, regardless of which oncogene was used (Fig. 2B, C), even when the oncogene-bearing cells and the cells that have been hyperpolarized are on opposite ends of the body. Complete suppression of ITLS was not achieved, because our reagents had to be tittered down to avoid perturbing the normal developmental processes of the organism. Future work is needed to refine the strategy for optimal management of the bioelectric crosstalk that goes awry in cancer; it is likely that a more nuanced strategy, which manages spatial relationships at higher resolution, will be required in clinical practice. Another important area for future investigation is slow time-dependent variability of V_mem_, as we only explored continuously-acting reagents and it is possible that important patterning information is encoded in the time profile of slowly-changing bioelectrical states [37, 139-142]. Optogenetics – the use of light to regulate ion channels with high spatio-temporal specificity - [143, 144] is a promising technology for both of these directions.

Resting potential changes can result from genomic, transcriptional, or post-translation control of ion channels and pumps. Importantly, we obtained tumor suppression using either Cl^−^ or K^+^-based hyperpolarization, just as we showed previously for cell-autonomous tumorigenesis and metastatic activation by depolarization [43, 46, 60, 61]. This rules out signaling pathways triggered by specific ion channels or even limited to specific ion types. Thus, in complement to the gene-focused idea of specific ion channels being intrinsic oncogenes or tumor suppressors [44, 48, 145], we suggest that a systems-level physiological property – resting potential – can be a powerful causal factor in regulating these processes.

### CLIC1-dependent hyperpolarization: a native target underlying suppression

A large body of literature reveals differential ion channel and transporter expression between tumor cells and their untransformed counterparts [146-150]. These native ion translocators are important not only as markers [52, 145, 151-153] but also as targets for interventions designed to manage the bioelectric state of cancer and surrounding tissue without needing gene therapy with heterologous channels. Exploiting native channels may allow manipulation of bioelectric signals merely by changing ionic composition of the cellular environment; indeed, we observed that hyperpolarization by high Cl^−^ reduces ITLS incidences (Fig. 3B) without additional channel misexpression. The alteration of tumorigenic transformation by ionic conditions in the medium is consistent with previous data showing an association between the (depolarizing) level of sodium and cancer [154-156], the control of cell differentiation by sodium levels [157-159] and pH [160], and more recent work on stem cell reprogramming by acid baths and streptolysin-mediated membrane permeability change [161]. Reprogramming of cell state by physiological cues is certainly of relevance to cancer [162-165]. However, two important novel aspects of our data are the focus on V_mem_ *per se*, not specific ions as obligate signals, and the non-local nature of the suppression mechanism.

A pharmacological loss-of-function experiment using chloride channel blockers (Fig. 3A) and a gene-specific dominant negative (Fig. 4) implicated chloride intracellular chloride channel 1 (CLIC1) as a likely candidate for the channel required for hyperpolarization in the long-range ITLS suppression. Roles for CLIC1 in carcinogenesis – albeit cell autonomous – have been shown previously in several studies. A search for ion channels in the cancer profiling database, Oncomine, turns up CLIC1 as one of the most upregulated genes [166], and chloride current associated with increased CLIC1 expression is present in progenitor cells isolated from human glioblastomas, and is responsible for promoting proliferation, clonogenicity, and tumorigenic capacity [167]. Channels like this are of high interest as a therapeutic modality [56], but it should be noted that it is not enough to look for blockers (a standard loss-of-function genetic strategy) – the key is to modulate V_mem_, which may mean opening or closing specific channels depending on the cells' surrounding milieu and its ion gradients. An understanding of V_mem_ as a regulatory agent may also explain the anti-cancer activity of a number of agents such as ivermectin [57], bafilomycin [168, 169], and salinomycin [170].

In *Xenopus*, CLIC1 is expressed throughout development, with progressive increase in expression from mid-blastula transition to the tadpole stages; spatially, CLIC1 transcripts are present primarily in the ectoderm and ectodermally-originated organs [94]. Interestingly, CLIC1 expression is missing from the gut region where we frequently observe ITLS on the ectoderm. Thus, CLIC1 proteins are located at a distance from oncogene-induced foci, showing that they can act like the overexpressed potassium channels – in long-range hyperpolarization. By overexpressing wildtype *Xenopus* CLIC1 and raising extracellular chloride levels, we are able to achieve higher suppression of oncogenic transformation of cells located at a distance. Moreover, this suppression can be blocked – despite the presence of high extracellular chloride levels – by introducing a dominant negative chloride channel mutant away from oncogene-expressing cells (Fig. 4). Interestingly, another study from our lab has documented the endogenous presence of glycine-gated channels: though not present in melanocytes their depolarization confers highly proliferative and metastatic phenotype in melanocytes in a long-range, serotonergic signaling pathway [60, 61].

### Transducing hyperpolarization into cell responses: HDAC1 inhibition

How do resting potential changes such as hyperpolarization impact on the transcriptional and epigenetic pathways of cancer? Recent studies have identified several mechanisms that transduce bioelectric signals at the cell membrane into biochemical responses (reviewed in [63, 104, 171]. Using a pharmacological suppression and molecular loss of function (Fig. 5A, B), we identified possible roles for gap junction communication (to be reported in a forthcoming study) and HDAC1 inhibition as signaling elements required for the long-range ITLS suppression. The introduction of a dominant negative HDAC1 mRNA [102] in a long-range manner was able to significantly increase neoplastic conversions of oncogene-expressing distant cells. The results are surprising relative to the well-known function of HDAC inhibitors as having antitumor activities, including reduced proliferation [172], increased differentiation/apoptosis [173], and that a number of them are on the clinical development pipeline for anti-cancer therapeutics [174-177]. In fact, variants of well-known HDAC inhibitors, such as vorinostat, (Merck) and romidepsin (Celgene) have already been developed and approved by the Food and Drug Administration for treating cutaneous T-cell lymphoma [178, 179]. However, consistent with our data, HDAC1 knock-down promotes early tumorigenesis in oncogene expressing cells [180], and genomic instability in a given cell can have a system-wide effect mediated by the immune system and other factors [181, 182]. Overall, this is consistent with our proposal that what is disrupted in cancer is a specific and highly modulated pattern of resting potentials - it is likely that the desired signaling cannot be achieved by universally increasing “anti-cancer” gene products. Depending on other factors that vary widely in different systems (such as ion concentrations, butyrate availability, and spatial arrangement of tumor and tissue), disbalance of voltage-dependent signaling in either direction could promote or suppress tumorigenesis.

We sought to understand how V_mem_ changes and acetylation state of chromatins could control tumor growth in a non-cell autonomous manner. Given the link between V_mem_-guided butyrate transport and HDAC activities the control cell behavior during regeneration [62] and local tumorigenesis [43], we tested the effect of targeting butyrate-producing bacteria on ITLS formation. Both novobiocin, an antibiotic against gram-positive bacteria, and a cocktail of antibiotics (gentamicin, metrodinazole, vancomycin, and clindamycin, data not shown) that have been verified to reduce levels of butyrate, significantly increased the rate of ITLS formation (Fig. 6). Our data are consistent with, and provide a novel mechanism explaining, several studies that investigated the effects of antibiotics on the gut microbiota, including butyrate-producing bacteria species, and their subsequent implications in increased cancer risks and incidences [183-186].

Our analysis was limited in two main ways: the lack of a technology for monitoring location of butyrate *in vivo*, and the inability to isolate and culture butyrate producing bacteria (which are highly anaerobic and do not grow under standard culture conditions [187]). Future work using GFP-expressing strains of appropriately engineered bacterial model species will further refine our understanding of the role of native microbiota on cancer progression and bioelectric signaling.

**Figure 7:**
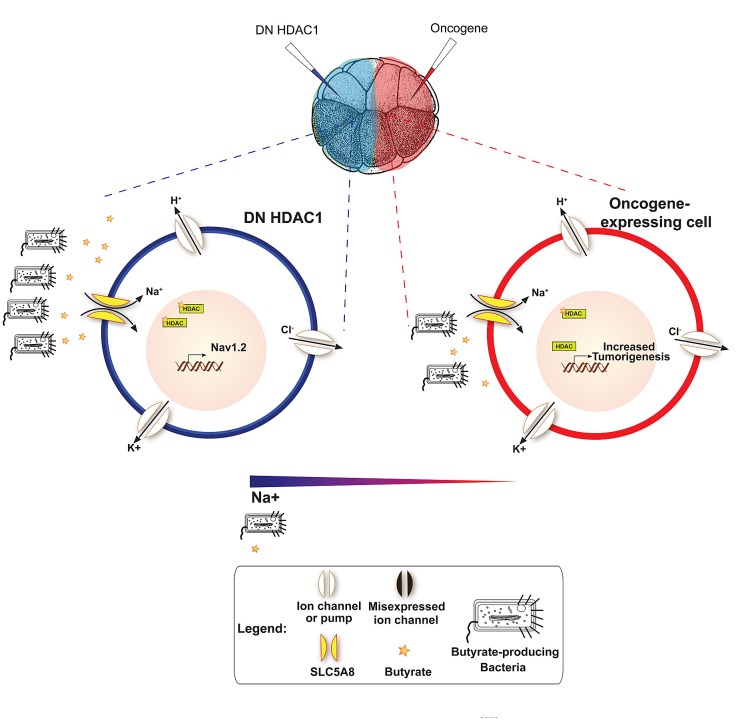
A model for V – mediated, HDAC1-dependent, long-range control of cell dynamics during neoplastic transformation It is known that HDAC1 inhibition leads to the altered transcriptional of regulation of several ion channel genes including NaV 1.2 [197] – a voltage gated sodium channel encoding gene One model consistent with our data is that the resulting Na^+^ gradient (more positive charge) would attract butyrate producing bacteria away from oncogene expressing cells (illustrated using *Escherichia coli* in Supp. Fig 1). Gram-positive bacteria are necessary for the production of butyrate, which gets imported into cells via SLC5A8 to reduce proliferation rate by inducing cell cycle arrest in tumor cells. Driving these bacteria away from ITLS cells would reduce the number of butyrate molecules available near oncogene cells, thus blocking ITLS suppression.

Collectively, the data presented here suggest a possible model (Fig. 7). Pharmacologically/molecular-genetically hyperpolarized cells have membrane that is negative on the inside, which creates a positively-charged cell surface environment. While bacteria – whether gram-positive or gram-negative – readily attach to a positively-charged surface, their viability is greatly diminished due to strong electrostatic interaction [188, 189]. As a result, distribution of butyrate producing-bacteria is skewed away from hyperpolarized cells in favor of oncogene-expressing cells, which are known to be depolarized pre-neoplastic transformation [43]. Availability of butyrate to these prospective tumor sites leads to HDAC inhibition and increased hyperacetylation of histones, which promote cell cycle arrest, leading to reduced rates of proliferation, apoptosis, and differentiation of cancer cells lines [105-107].

While the spatial dynamics of butyrate and bacteria remain to be investigated, our data reveal a novel long-range pathway regulating oncogene-mediated tumorigenesis at a distance. We do not yet know the maximum extent, in a large organism, of such long-range signaling by hyperpolarization. Future work must also examine the roles of remote endogenous bioelectric states in mammalian cancer models; if conserved, these data suggest a number of detection and treatment modalities focused on V_mem_ modulation. Because such strategies could be designed using ion channel drugs [190, 191] already approved for human use (as anti-epileptic agents), exciting opportunities for biomedicine may be presented by investigations of bioelectric signaling in cancer at a level beyond that of single cells.

## MATERIALS AND METHODS

### Animal Husbandry

*Xenopus laevis* embryos were collected and fertilized *in vitro* according to standard protocols [192], in 0.1X Modified Marc's Ringers (MMR; pH 7.8) with 0.1% Gentamicin. *Xenopus* embryos were housed at 14-18°C and staged according to Nieuwkoop and Faber [193]. All experimental procedures involving the use of animals for experimental purposes were approved by the Institutional Animal Care and Use Committees (IACUC) and Tufts University Department of Lab Animal Medicine (DLAM) under the protocol number M2011-70.

### Microinjection

Fertilized *Xenopus* embryos were transferred into mesh-bottomed dishes with 3% Ficoll and injected with capped, synthetic mRNAs (made using the Ambion Message Machine kit) dissolved in water at the stages indicated. 2 hours post injection, embryos were transferred into 0.75× MMR for 45 minutes before they were washed and cultured in 0.1X MMR until desired stage was reached. Constructs used included: FUCCI (*f*luorescent *u*biquitination-based *c*ell *c*ycle *i*ndicator) pair: mKO2-Cdt1 and mAG-Geminin [71, 72]; Gli1 [68], Xrel3 [194], *KRAS*^G12D^ [69], and Kv1.5 [86]; CLIC1-2A-GFP3 and CLIC1C24S-tdTomato; DN-HDAC1 [102].

### Drug treatments

Embryos were exposed in 0.1× MMR for the stages indicated to: Anthracene-9-Carboxylic Acid (ACA) 67 μM; indanyloxyacetic acid (*IAA*-94), 55 μM; 5-Nitro-2-(3-phenylpropylamino) benzoic acid (NPPB, Tocaris Bioscience, Bristol, UK), 8.5 nM; nalidixic acid 43 μM; novobiocin sodium salt 78 μM; gentamicin, 20 μM; metrodinazole, 20 μM; vancomycin hydrochloride (Santacruz Biotechnology, Texas, USA) 20 μM; Choline chloride, 70mM and clindamycin hydrochloride, 20 μM. All compounds were obtained from Sigma-Aldrich, St. Louis, MO unless otherwise noted.

### Immunohistochemistry

Spatial analysis of leukocyte presence was performed by immunohistochemistry in paraffin sections, using an anti-XL2 antibody [195]. Briefly, embryos were fixed overnight in MEMFA [192], embedded in paraffin and sectioned at 5 μm using a Leica microtome. After deparaffinizing and rehydrating, tissue sections were permeabilized in phosphate buffered saline (PBS) + 0.1X Triton X-100 for 30 minutes, blocked with 10% goat serum in PBS + 0.1% tween-20 for 1 hour, and incubated at 4°C overnight with anti-XL2 primary antibody. Sections were then washed six times with PBST (30 minutes each at room temperature) and incubated with Alexa-Fluor-555-conjugated secondary antibody at 1:1000 in PBST + 10% goat serum overnight at 4°C. After five 30-minute washes in PBST, sections were mounted on a slide and photographed using the TRITC filter set on an Olympus BX61 spinning-disk confocal microscope with Hamamatsu ORCA digital CCD camera.

### Hypoxia detection

Immunochemical detection of tissue hypoxia was performed by immunoperoxidase reaction in paraffin sections, using the Hypoxy™-1 Plus Kit (Hypoxyprobe, Inc, MA, USA). St.34 embryos with *KRAS*^G12D^ ITLS were incubated in 300 µM of pimonidazol HCl (Hypoxyprobe™-1). Paraffin sections were prepared for immunostaining as described above. Tissue sections were then incubated using 1:50 dilution of FITC-MAB1, which binds to protein adducts of pimonidazole in hypoxic regions, and counterstained with 1:50 dilution of horseradish peroxidase conjugated anti-FITC secondary.

### pH measurements

pH measurements were calculated from BCECF fluorescent signals as described previously in [196]. Briefly, embryos were incubated in a 5 µM BCECF, AM (2',7'-Bis-(2-Carboxyethyl)-5-(and-6)-Carboxyfluorescein, Acetoxymethyl Ester (Life Technologies, NY, USA) solution. Excess dye was washed out, and embryos were anesthetized with MS222. To image BCECF, a dual excitation dye, filters were EX 450/20, D 460, EM 535/30 (the isobestic point) and EX 500/20, D 515, EM 535/30. To calibrate BCECF in our system, a prototonophore, CCCP, and NaOH were used to drive pH values to 4 (limiting minimum) and 9 (limiting maximum), respectively, by observing gradual decrease and increase of fluorescence. A plot profile of pixel intensities was then taken from ITLS and control regions, and conversions to pH values were made as described in [196]

### Nuclear morphometry

Sections taken through ITLS and control regions were stained with Hoechst Blue (Life Technologies, NY, USA), imaged using the DAPI filter set on an Olympus BX61 spinning-disk confocal microscope with Hamamatsu ORCA digital CCD camera, and analyzed by using ImageJ. The threshold and analyze particle tools in ImageJ were used to interactively outline each nucleus and determine its size and shape.

### Statistical analysis

All statistical analyses were performed using GraphPad InStat v. 3.10 (GraphPad Software, La Jolla, CA, USA). Data were expressed as the mean unless otherwise noted. Error bars represent standard error. The differences between treatment groups were analyzed using Student's *t*-test, Chi-squared test or One-way ANOVA, (tukey's post hoc comparisons), and the null hypothesis was rejected at the 0.05 level.

## SUPPLEMENTARY FIGURE


