# Supplementary material for: Transmembrane voltage potential of somatic cells controls oncogene-mediated tumorigenesis at long-range
Source: Oncotarget. 2014 May 1;5(10):3287–306. doi: 10.18632/oncotarget.1935 (PMC4102810; doi:10.18632/oncotarget.1935)
Supplement: Supplementary file 1 [file oncotarget-05-3287-s001.pdf]

Transmembrane voltage potential of somatic cells controls oncogene-mediated tumorigenesis at long-range

SUPPLEMENTAL MATERIAL

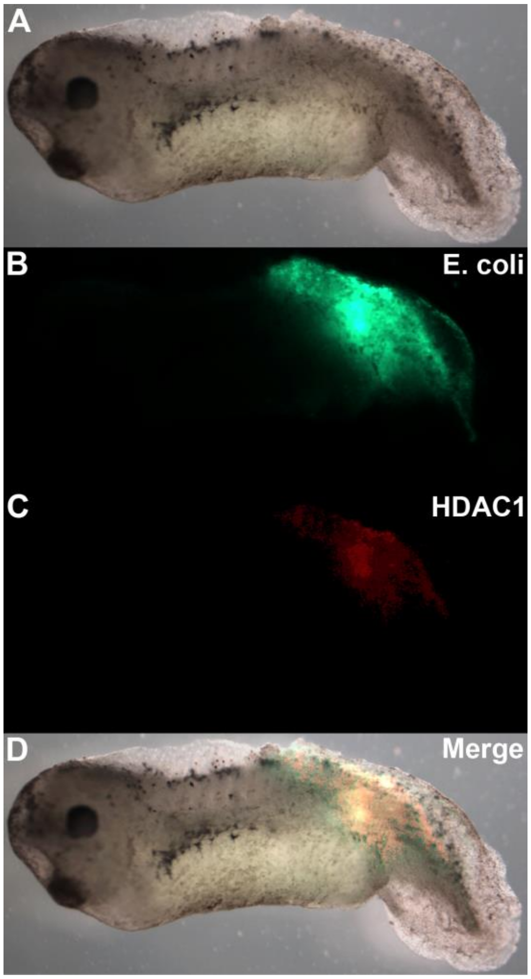

Supplemental figure 1: E. coli preferentially colonize DN-HDAC1 expressing cells.

(A) Brightfield image of DN-HDAC1/lineage tracer injected (into 1 cell of 16 cell stage) embryo, which is also infected with GFP-expressing *E. coli* at stage 12.

(B) *E. coli* colonizes region of the tail marked by GFP expression.

(C) The same region colonized by *E. coli* is also the site of DN-HDAC1 expression, which is represented by the red lineage tracer.

(D) Merged photo of B & C shows that site of HDAC1 expression matches the site of *E. coli* colonization.
